# Comparison of the effects of different doses of Glucocorticoids on distinct subtypes of Guillain-Barré syndrome in Southern China

**DOI:** 10.1186/s12883-022-02567-8

**Published:** 2022-02-05

**Authors:** Linzhuo Ma, Shuping Liu, Zheman Xiao, Jingxia Guan, Yin Liu, Jiajia Yao, Zuneng Lu

**Affiliations:** Department of Neurology, Renmin hospital of Wuhan University, 99 Zhang Zhidong Road, Wuchang District, Hubei Province 430060 Wuhan, P. R. China

**Keywords:** Guillain-Barré syndrome, Glucocorticoids, Southern China, subtype

## Abstract

**Background:**

The effect of Glucocorticoids (GCs) on the treatment of Guillain-Barré syndrome (GBS) has been controversial. There is no information on whether specific subtypes of GBS respond differently to GCs. In this setting, we aimed to discuss whether GCs treating yield different effects in the distinct subtypes (acute inflammatory demyelinating polyneuropathy, AIDP; acute motor axonal neuropathy, AMAN). And further, we analyzed the impact of different doses on the outcome.

**Methods:**

Medical records of 448 patients with a diagnosis of classic GBS admitted to 31 tertiary hospitals, located in 14 provinces of Southern China, from 1 January 2013 to 30 September 2016, were retrospectively collected. And 251 patients treated with GCs alone (AIDP=189, AMAN=62) were reviewed and analyzed.

**Results:**

After GCs treatment, the Hughes score of AIDP patients was significantly lower than that of AMAN patients at discharge (*P*=0.005) and 3 months after onset (*P<*0.001). Further analysis revealed that among AIDP patients, the high-dose group had significantly shorter hospital stay (*P*=0.023), lower Hughes score at nadir (*P<*0.001), at discharge (*P*=0.005), and 3 months after onset (*P<*0.001), compared with the low-dose group. However, for AMAN patients, the outcome difference between groups was nonsignificant.

**Conclusion:**

Our data suggest that the high doses of GCs may result, at least in part, from the side of the duration of hospital stay and short-term outcome, favorable outcomes in AIDP patients. Therefore, we cannot completely deny the priority of GCs in the treatment of GBS, because the effect of different doses of GCs varies in treating different subtypes. More studies are needed in the future to further validate this issue.

**Trial registration:**

ChiCTR-RRC-17014152. Registered 26 December 2017- Retrospectively registered.

## Introduction

Guillain-Barré syndrome (GBS) is an immune-mediated acute peripheral neuropathy involving mainly spinal nerve roots, peripheral nerves and cerebral nerves, and is currently the most common cause of acute flaccid paralysis worldwide. As an autoimmune disease (AID) with a high rate of mortality and disability, immunotherapy is essential [1, 2].

Glucocorticoids (GCs) is considered as the most commonly used drug for the treatment of AID worldwide because of its cost-effectiveness and strong immunosuppressive effect [3]. Unfortunately, its use in GBS patients is controversial [4, 5]. Clinical trials in Europe and North America did not observe significant efficacy of GCs alone in GBS, however, as it currently stands, the actual efficacy of GCs may be underestimated, because these above-mentioned studies did not discuss the efficacy of GCs in different subtypes in a categorical manner, and the use and dosage of GCs were not uniform [6–8]. Scholars such as Hughes have suggested that patients with GBS with conduction block respond well to GCs, while the use of GCs in patients with denervation delays the recovery of GBS, although the specific mechanism needs to be further explored [9].

According to neuroelectrophysiological studies, GBS consists of two major subtypes, acute inflammatory demyelinating polyneuropathy (AIDP), and acute motor axonal neuropathy (AMAN) [10]. AIDP is associated with macrophage and CD4^+^ T cell-mediated inflammation and peripheral nerve demyelination, whereas AMAN is mainly associated with the involvement of ganglioside autoantibodies and complement [11]. Given that these two major subtypes have different pathological characteristics and pathogenesis, and their epidemiology in Asia differs from foreign studies, it is necessary to explore the mechanism of action and effects of GCs based on different subtypes.

## Methods

### Patient ascertainment

**Fig. 1:**
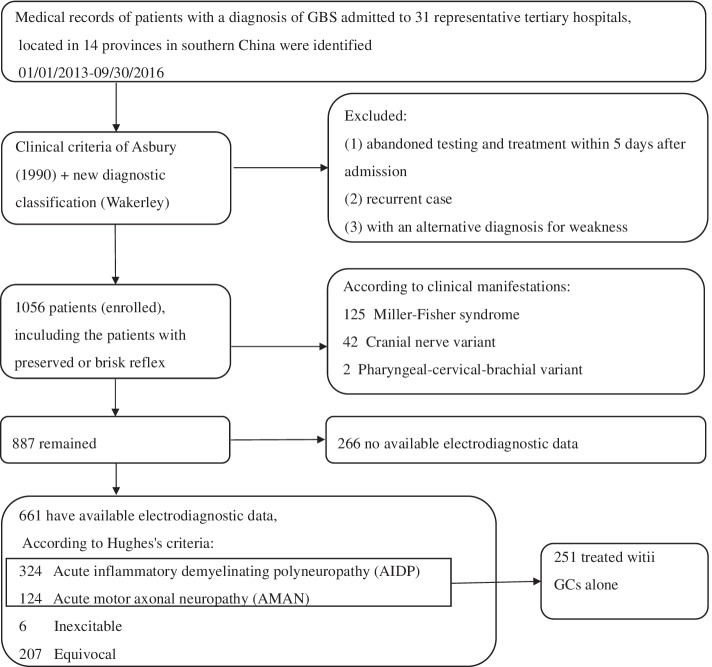
Flow chart of patient ascertainment

This is a retrospective multicenter study and the medical records of consecutive hospitalized patients with a diagnosis of GBS in 31 representative tertiary hospitals, located in 14 provinces in southern China, between 1 January 2013 and 30 September 2016, were collected. Patients who fulfilled the established clinical criteria of Asbury and Cornblath (1990) were enrolled [12]. In addition, the patients whose clinical presentation and ancillary data were typical of GBS except for preservation or exaggeration of reflexes were also included. Details regarding clinical data extraction and analysis, including inclusion and exclusion criteria, were described in our previous study [13]. Those patients with a diagnosis of AIDP or AMAN, including acute motor and sensory axonal neuropathy (AMSAN), and treated with GCs alone and symptomatic supportive treatment were analyzed (Figure 1). The study was approved by the ethics committee of the Renmin Hospital of Wuhan University, and the need for informed consent was waived.

### Treatment Grouping

The high dose group patients received methylprednisolone (MPS) (250–1,000 mg/d) for 3-5 days and then tapered as clinically indicated; while the low dose group received MPS (40–120 mg/d) for 3-5 days, or dexamethasone (10-20mg/d) for 5-7 days followed by a tapered dosage, or else oral prednisolone at 1 mg/kg/day for 1 week, tailed off over the next 2 months in a similar manner.

### Information Extraction

Information on age, sex, preceding events, initial symptoms, concomitant symptoms, severity at admission, at nadir, at discharge, length of hospitalization, findings of electrodiagnosis (EDX), treatment regime, types and doses of GCs were extracted. The motor function deficits of included patients were assessed by the Hughes Functional Grading Scale, a widely accepted scale of disability for GBS (grade 6, dead; grade 5, requiring assisted respiration; grade 4, bed-bound; grade 3, able to walk with aid; grade 2, able to walk independently; grade 1, minimal signs and symptoms, able to run; grade 0, normal) [14]. Details regarding clinical data extraction were described in our previous study [13].

### Statistical analysis

Statistical analysis was performed using IBM SPSS 23.0 software. Categorical data were presented as proportions, and continuous data were presented as mean ± standard deviation (SD). Differences in proportions were tested by the χ2 tests. The continuous variables with a normal distribution were tested using the Student’s t-test or analysis of variance test, and the continuous variables with a skewed distribution were tested using the Mann-Whitney U test or the Kruskal-W allis analysis. For all statistical tests, *P*<0.05 was considered to be significant.

## Results

### Baseline clinical characteristics

Finally, 251 patients with a diagnosis of GBS, including 189 (75.3%) cases with AIDP and 62 (24.7%) cases with AMAN, were analyzed. Among whom 157 (62.5%) were men and 94 (37.5%) were women, and 168 (66.9%) patients came from rural areas. The mean age was 49.6 years (age range 17–83 years). 133(53.0%) patients were treated with high-dose GCs, who received intravenous methylprednisolone (≥250 mg) for 3-5 days, followed by gradual reduction to oral prednisone. 118 (47.0%) patients were treated with low-dose GC, including dexamethasone, prednisone, and low-dose methylprednisolone (< 250 mg). Two patients died during their hospital stay. The common autonomic symptoms of our patients included hypertension, cardiac arrhythmia, hypotension, orthostatic hypotension, sweating, bowel and bladder incontinence or retention.

### Effects of GCs on the treatment of different subtypes (AIDP *vs* AMAN)

There was no statistically significant difference between the two groups in terms of certain baseline characteristics, such as age, gender, urban-rural distribution, Hughes score on admission. As to complications and concomitant symptoms, autonomic dysfunctions and laboratory abnormality, for example, we found no significant difference between the two groups. However, facial/bulbar paralysis (45.5 *vs* 16.1, *P<*0.001) and paresthesia (49.2 *vs* 32.3, *P*=0.027) were more frequently observed in patients with AIDP, in whom hyperreflexia occurred less frequently (5.8 *vs* 19.3, *P*=0.004). With regard to clinical outcomes, AIDP patients had a significantly lower Hughes score at discharge (2.51±0.98 *vs* 2.84±0.73, *P*= 0.005) and 3 months after onset (2.06±1.14 *vs* 2.50±0.80, *P<*0.001), compared to that in AMAN patients (Table 1).

**Table 1:** Effects of GCs on the treatment of different subtypes

Parameters	AIDP (*n*=189)	AMAN (*n*=62)	*P* value (two-tailed)
Age (years)	49.88±18.07	48.61±17.46	0.631
Male, n (%)	123(65.1)	34(54.9)	0.174
Rural area, n (%)	125(66.1)	43(69.4)	0.756
Hughes score on admission (g)	2.97±0.93	3.10±0.88	0.451
Hughes score at nadir (g)	3.50±0.89	3.71±0.69	0.155
Neurological symptoms, n (%)			
Facial/bulbar paralysis	86(45.5)	10(16.1)	<0.001*
Oculomotor paralysis	14(7.4)	3(4.8)	0.771
Paresthesia	93(49.2)	20(32.3)	0.027*
Hyperreflexia	11(5.8)	12(19.3)	0.004*
Complication, n (%)			
Autonomic dysfunction, n (%)			
Hypertension	38(20.1)	11(17.7)	0.854
Cardiac arrhythmia	13(6.9)	7(11.3)	0.283
Urinary retention	21(11.1)	12(19.4)	0.128
Deep venous thrombosis	7(3.7)	3(4.8)	0.712
Dyspnoea	49(25.9)	10(16.1)	0.124
Pulmonary infection	28(14.8)	8(12.9)	0.836
Diabetes	16(8.5)	4(6.5)	0.789
Laboratory abnormality, n (%)			
Hyponatraemia	53(28.0)	13(16.7)	0.320
Hypokalemia	42(22.2)	14(22.6)	1.000
Hospital stay (days)	14.18±8.10	15.35±7.29	0.221
Mechanical ventilation, n (%)	19(10.1)	7(11.3)	0.811
Death in hospital stay, n (%)	1(0.5)	1(1.6)	0.434
Hughes score at discharge (g)	2.51±0.98	2.84±0.73	0.005*
Hughes score of 3 months after onset (g)	2.06±1.14	2.50±0.80	<0.001*

Values are mean ± standard deviation unless otherwise specified

GCs Glucocorticoids, AIDP acute inflammatory demyelinating polyneuropathy, AMAN acute motor axonal neuropathy axonal neuropathy

* Significant difference between groups at *p* < 0.05

### Effects of high-dose and low-dose GCs on the treatment of AIDP patients

A total of 189 AIDP patients were enrolled in this study, 98 (51.9%) of them were treated with high-dose GCs and 91 (48.1%) patients received low-dose GCs. There was no statistically significant difference between the two groups in terms of age, gender, urban-rural distribution, and Hughes score on admission. Notably, patients that received low-dose GCs had a higher frequency of pulmonary infection (9.2 *vs* 20.9, *P*=0.026). Regarding to the clinical outcomes, patients in the high-dose group had a significantly shorter hospital stay (13.27±8.47 *vs* 15.16±7.62, *P*=0.023), lower Hughes score at nadir (3.28±0.88 *vs* 3.74±0.74, *P<*0.001), at discharge (2.36±1.03 *vs* 2.68±0.88, *P*=0.005) and 3 months after onset (1.83±1.30 *vs* 2.30±0.89, *P<*0.001), when compared with the low-dose group. During the hospitalization, one patient died (Table 2).

**Table 2:** Effects of high-dose and low-dose GCs on the treatment of AIDP

Parameters	High-dose(n=98)	Low-dose(n=91)	P value (two-tailed)
Age (mean, years)	48.49±17.94	51.85±17.37	0.286
Male, n (%)	65(66.3)	58 (63.7)	0.761
Rural area, n (%)	62(63.3)	63(69.2)	0.443
Hughes score on admission (mean, g)	2.99±0.91	2.96±0.94	0.909
Hughes score at nadir (mean, g)	3.28±0.88	3.74±0.74	<0.001*
Neurological symptoms, n (%)			
Facial/bulbar paralysis	47(47.8)	39(42.9)	0.559
Oculomotor paralysis	6(6.1)	8(8.8)	0.583
Paresthesia	42(42.9)	51(56.0)	0.081
Hyperreflexia	6(6.1)	5(5.5)	1.000
Complication, n (%)			
Autonomic dysfunction			
Hypertension	17(17.3)	21(23.1)	0.413
Cardiac arrhythmia	6(6.1)	7(7.7)	0.777
Urinary retention	9(9.2)	12(13.2)	0.488
Deep venous thrombosis	3(3.1)	4(4.4)	0.713
Dyspnoea	28(28.6)	21(23.1)	0.411
Pulmonary infection	9(9.2)	19(20.9)	0.026*
Diabetes	9(9.2)	7(7.7)	0.797
Laboratory abnormality, n (%)			
Hyponatraemia	22(22.4)	31(34.1)	0.105
Hypoalbuminaemia	19(19.4)	23(25.3)	0.383
Hospital stay (days)	13.27±8.47	15.16±7.62	0.023*
Mechanical ventilation, n (%)	11(11.2)	8(8.8)	0.635
Death in hospital stay, n (%)	1(1.0)	0	-
Hughes score at discharge (g)	2.36±1.03	2.68±0.88	0.005*
Hughes score of 3 months after onset (g)	1.83±1.30	2.30±0.89	<0.001*

Values are mean ± standard deviation unless otherwise specified

GCs Glucocorticoids, AIDP acute inflammatory demyelinating polyneuropathy

* Significant difference between groups at p < 0.05

### Effects of high-dose and low-dose GCs on the treatment of AMAN patients

Totally, 62 patients with AMAN were enrolled in our study and received different treatment doses of GCs. No differences in baseline characteristics between groups were statistically significant. Concerning the short-term outcome, such as hospital stay (*P*=0.943), Hughes score at nadir (*P*=0.262), Hughes score at discharge (*P*=0.591) and Hughes score at 3 months after onset (*P*=0.386), the differences between the two groups were non-significant (Table 3).

**Table 3:** Effects of high-dose and low-dose GCs on the treatment of AMAN

Parameters	High-dose (*n*=35)	Low-dose (*n*=27)	*P* value (two-tailed)
Age (years)	49.31±15.69	47.70±19.80	0.722
Male, n (%)	20(51.4)	14(51.9)	0.798
Rural area, n (%)	26 (74.3)	17(63.0)	0.805
Hughes score on admission (g)	3.11±0.93	3.07±0.83	0.940
Hughes score at nadir (mean, g)	3.63±0.69	3.81±0.68	0.262
Neurological symptoms, n (%)			
Facial/bulbar paralysis	4(11.4)	6(22.2)	0.308
Oculomotor paralysis	2(5.7)	1(3.7)	1.000
Paresthesia	12(34.3)	8(29.6)	0.788
Hyperreflexia	7(20.0)	5(18.5)	1.000
Complication, n (%)			
Autonomic dysfunction			
Hypertension	6(17.1)	5(18.5)	0.735
Cardiac arrhythmia	4(11.4)	3(11.1)	1.000
Urinary retention	5(14.3)	7(25.9)	0.335
Deep venous thrombosis	1(2.9)	2(7.4)	0.575
Dyspnoea	4(11.4)	6(22.2)	0.308
Pulmonary infection	3(8.6)	5(18.5)	0.279
Diabetes	3(8.6)	1(3.7)	0.626
Laboratory abnormality, n (%)			
Hyponatraemia	6(17.1)	7(25.9)	0.532
Hypoalbuminaemia	9(25.7)	5(18.5)	0.555
Hospital stay (days)	15.66±8.11	14.96±6.20	0.943
Mechanical ventilation, n (%)	3(8.6)	4(14.9)	0.689
Death in hospital stay, n (%)	0	1(5.00)	-
Hughes score at discharge (g)	2.80±0.76	2.89±0.70	0.591
Hughes score of 3 months after onset (g)	2.43±0.74	2.59±0.89	0.386

Values are mean ± standard deviation unless otherwise specified

*GCs* Glucocorticoids, *AMAN* acute motor axonal neuropathy axonal neuropathy

## Discussion

Our first multi-center study showed that the Hughes score at discharge and 3 months after onset were significantly lower in AIDP patients treated with GCs compared to that in AMAN patients. Further analysis found that among AIDP patients, the high-dose group had shorter hospitalization days and significantly lower Hughes score at nadir, at discharge and 3 months after onset than that in the low-dose group. However, among AMAN patients, according to our data, the short-term outcome in the high-dose group was not significantly different from that in the low-dose group.

In regarding to complications, we found that, among AIDP patients, the incidence of pulmonary infections was higher in the low-dose group, which we speculated that the longer hospital stay of patients in the low-dose group may account. Because, as the length of hospital stay increases, says from some kind of significance, the effective activity of patients decreases and the risk of pathogenic bacteria infection greatly increases [15]. On the other side, studies have demonstrated that patients with refractory pulmonary treated with high-dose corticosteroid could achieve defervescence earlier and have a shorter hospitalization [16].

These data above suggest that we can’t dismiss completely the role of GCs in the treatment of GBS, subtyping to explore the effects of different doses of GCs on GBS treatment is necessary. After all, in China, especially in the 1990s, GCs were the drug of choice in the treatment of GBS because of their civilian price, and clinical observations found good results in many patients [17].

A study used a rabbit model of the axonal form of GBS initially explored the reasons for the ineffectiveness of GCs in treating AMAN, suggesting that MPS did not reduce complement C3 deposition and sodium (Nav) channel disruption, but significantly reduced macrophage infiltration in the ventral roots and thus delay the axonal regeneration [18]. Studies of pathophysiology about AMAN have shown that the invasion of macrophages was rare at the acute progressive phase but significantly more frequent at the site of inflammation mainly during the recovery phase, which suggested a role for macrophages in the clearance of damaged myelin and axon fragments and promoting nerve repair and regeneration [19]. Whereas, the classical experimental autoimmune neuritis (EAN) model, which highly replicates human AIDP in terms of clinical manifestations, immunology, histopathology, and electrophysiology [20], indicated that “Classically” activated (M1) macrophages mainly accumulated at the acute phase of EAN and promoted the inflammatory response, while during the recovery phase, macrophages could change their expression profile, M2 macrophages attenuated inflammation and promoted tissue repair [21, 22]. Ultrastructural studies showed that macrophage-mediated nerve injury was a pathological hallmark of AIDP/EAN [21–23]. Macrophages (M1) were involved in this process by regulating cytokines, chemokines, adhesion molecules, nitric oxide (NO) and matrix metalloproteinases (MMPs), and as major antigen-presenting and effector cells, macrophages played a key role in EAN pathogenesis by expressing antigens and promoting Th1 and Th17 polarization [24].

In summary, we hypothesize that the different mechanisms of macrophages' role in the inflammatory response of AIDP and AMAN may lead to different effects of GCs therapy. We will further test our hypothesis through animal experiments.

As a multicenter study, we derived relatively powerful results, but there exist inevitably some limitations. First, as a retrospective study, the long-term follow-up information was insufficient to further explore the prognosis of patients with different subtypes treated with different doses of GCs, further studies were anticipated; Second, the number of patients with AMAN subtypes in this study was relatively small; Third, because the study was a retrospective review of medical records and database, extracting bias was unavoidable. However, in order to reduce the bias as much as possible, a unified parameter standard in the analysis of NCS was adopted and data were extracted by our team members through strict training.

## Conclusion

In conclusion, our data firstly provides information about whether the responses to GCs differ between the principal subtypes of GBS, and prompts recommendations about the design of future GBS trails. GCs induce different effects in specific GBS subtypes, among which high-dose GCs therapy has a better prognosis for patients with AIDP. The effects of GCs on GBS subtypes should be discussed separately in future clinical trials to explore its mechanism of action and provide more timely and effective treatment measures for GBS patients.

## Data Availability

The datasets used and/or analyzed during the current study are available from the corresponding author on reasonable request.

## Abbreviations

GCs: Glucocorticoids
GBS: Guillain-Barré syndrome
AIDP: Acute inflammatory demyelinating polyneuropathy
AMAN: Acute motor axonal neuropathy
AMSAN: Acute motor and sensory axonal neuropathy
AID: Autoimmune disease
MPS: Methylprednisolone
SD: Standard deviation
EAN: Experimental autoimmune neuritis
NO: Nitric oxide
MMPS: Matrix metalloproteinases

## References

[1] van den Berg B, Walgaard C, Drenthen J, Fokke C, Jacobs BC, van Doorn PA (2014). Guillain-Barré syndrome: pathogenesis, diagnosis, treatment and prognosis. Nat Rev Neurol..

[2] Sipilä JOT, Soilu-Hänninen M, Ruuskanen JO, Rautava P, Kytö V (2017). Epidemiology of Guillain-Barré syndrome in Finland 2004–2014. J Peripher Nerv Syst..

[3] Straub RH, Cutolo M. Glucocorticoids and chronic inflammation. Rheumatology (Oxford).2016;55(suppl 2):ii6–14. 10.1093/rheumatology/kew348. 10.1093/rheumatology/kew348 27856655

[4] Hughes RA (2004). Treatment of Guillain-Barré syndrome with corticosteroids: lack of benefit?. Lancet..

[5] Mastaglia FL (2005). Neuromuscular disorders: molecular and therapeutic insights. Lancet Neurol..

[6] Walgaard C, Lingsma HF, Ruts L, van Doorn PA, Steyerberg EW, Jacobs BC (2011). Early recognition of poor prognosis in Guillain-Barre syndrome. Neurology..

[7] Double-blind trial of intravenous methylprednisolone in Guillain-Barré syndrome. Guillain-Barré Syndrome Steroid Trial Group. Lancet. 1993;341(8845):586–90. 8094828

[8] van Koningsveld R, Schmitz PI, Meché FG, Visser LH, Meulstee J, van Doorn PA, Dutch GBS study group. Effect of methylprednisolone when added to standard treatment with intravenous immunoglobulin for Guillain-Barré syndrome: randomized trial. Lancet. 2004;363(9404):192–6. 10.1016/s0140-6736(03)15324-x. 10.1016/s0140-6736(03)15324-x 14738791

[9] Hughes RA, Brassington R, Gunn AA, van Doorn PA. Corticosteroids for Guillain-Barré syndrome. Cochrane Database Syst Rev. 2016;10(10):CD001446. 10.1002/14651858.CD001446.pub5. 10.1002/14651858.CD001446.pub5 PMC6464149 27775812

[10] Malek E, Salameh J (2019). Guillain-Barre Syndrome. Semin Neurol..

[11] Fan X, Zhang H, Cheng Y, Jiang X, Zhu J, Jin T (2016). Double Roles of Macrophages in Human Neuroimmune Diseases and Their Animal Models. Mediators Inflamm..

[12] Asbury AK, Cornblath DR. Assessment of current diagnostic criteria for Guillain-Barré syndrome. Ann Neurol. 1990;27 Suppl:S21–4. 10.1002/ana.410270707. 10.1002/ana.410270707 2194422

[13] Liu S, Xiao Z, Lou M, Ji F, Shao B, Dai H (2018). Guillain-Barré syndrome in southern China: retrospective analysis of hospitalised patients from 14 provinces in the area south of the Huaihe River. J Neurol Neurosurg Psychiatry..

[14] Hughes RA, Newsom-Davis JM, Perkin GD, Pierce JM (1978). Controlled trial prednisolone in acute polyneuropathy. Lancet..

[15] Kuderer NM, Dale DC, Crawford J, Cosler LE, Lyman GH (2006). Mortality, morbidity, and cost associated with febrile neutropenia in adult cancer patients. Cancer..

[16] Okumura T, Kawada JI, Tanaka M, Narita K, Ishiguro T, Hirayama Y (2019). Comparison of high-dose and low-dose corticosteroid therapy for refractory Mycoplasma pneumoniae pneumonia in children. J Infect Chemother..

[17] Cheng Q, Wang DS, Jiang GX, Han H, Zhang Y, Wang WZ (2003). Prospective study of clinical epidemiology of Guillain-Barré syndrome in Harbin, China. J Neurol Sci..

[18] Wang YZ, Lv H, Shi QG, Fan XT, Li L, Yi Wong AH, Hao YL, Si CP, Li CL, Yuki N (2015). Action mechanism of corticosteroids to aggravate Guillain-Barré syndrome. Sci Rep..

[19] Susuki K, Rasband MN, Tohyama K, Koibuchi K, Okamoto S, Funakoshi K, Hirata K, Baba H, Yuki N (2007). Anti-GM1 antibodies cause complement-mediated disruption of sodium channel clusters in peripheral motor nerve fibers. J Neurosci..

[20] Gonsalvez DG, Fletcher JL, Yoo SW, Wood RJ, Murray SS, Xiao J (2017). A Simple Approach to Induce Experimental Autoimmune Neuritis in C57BL/6 Mice for Functional and Neuropathological Assessments. J Vis Exp..

[21] Shen D, Chu F, Lang Y, Geng Y, Zheng X, Zhu J, Liu K (2018). Beneficial or Harmful Role of Macrophages in Guillain-Barré Syndrome and Experimental Autoimmune Neuritis. Mediators Inflamm..

[22] Hartung HP, Schäfer B, Heininger K, Stoll G, Toyka KV. The role of macrophages and eicosanoids in the pathogenesis of experimental allergic neuritis. Serial clinical, electrophysiological, biochemical and morphological observations. Brain. 1988;111(Pt 5):1039–1059. 10.1093/brain/111.5.1039. 10.1093/brain/111.5.1039 2846115

[23] Kiefer R, Kieseier BC, Stoll G, Hartung HP (2001). The role of macrophages in immune-mediated damage to the peripheral nervous system. Prog Neurobiol..

[24] Han R, Xiao J, Zhai H, Hao J (2016). Dimethyl fumarate attenuates experimental autoimmune neuritis through the nuclear factor erythroid-derived 2-related factor 2/hemoxygenase-1 pathway by altering the balance of M1/M2 macrophages. J Neuroinflammation..

